# Research Progress on Anti-Aging with Natural Products: From Pathway Modulation to AI-Driven Discovery

**DOI:** 10.3390/biom15101384

**Published:** 2025-09-29

**Authors:** Chang Hyung Lee, Sang-Han Lee

**Affiliations:** 1 Harvard John A. Paulson School of Engineering and Applied Sciences, Harvard University, Allston, MA 02134, USA; changhyung@skku.edu; 2 College of Pharmacy, Sungkyunkwan University, Suwon 16419, Republic of Korea; 3 Bio-MAX Institute, Seoul National University, Seoul 08826, Republic of Korea; 4 Department of Food Science and Biotechnology, Kyungpook National University, Daegu 41566, Republic of Korea; 5 Food and Bio-Industry Research Institute, Inner Beauty/Antiaging Center, Kyungpook National University, Daegu 41566, Republic of Korea

**Keywords:** healthy aging, natural products, inflammation, autophagy, artificial intelligence, antioxidant defense

## Abstract

Aging results from the combined effects of oxidative stress, chronic low-grade inflammation, mitochondrial decline, and cellular senescence, which together drive age-related disorders. Natural products ranging from polyphenols and terpenoids to alkaloids, polysaccharides, peptides, and marine metabolites can influence central pathways such as Nrf2/ARE, NF-κB, MAPK, JAK/STAT, AMPK/PGC1-α, mTOR, and SIRT1/FOXO. By doing so, they strengthen antioxidant defenses, temper inflammation, preserve mitochondrial balance, and regulate autophagy. There is increasing attention to synergy, where combinations of bioactives can achieve stronger and more balanced effects than single agents alone. Advances in artificial intelligence are accelerating this discovery process, while greener extraction and smarter delivery systems such as deep eutectic solvents and nanostructured carriers are improving bioavailability and consistency. Together, these developments underscore the promise of natural product-based strategies for healthy aging. Grounded in rigor and reproducibility, this Special Issue aims to inspire translational advances toward healthier and more graceful aging.

## 1. Introduction: Aging and Natural Products

Aging is a multifactorial and inevitable biological process shaped by the interplay of genetic predisposition, environmental exposures, and lifestyle choices. At the cellular and systemic levels, aging manifests through hallmarks such as oxidative stress, chronic low-grade inflammation, mitochondrial dysfunction, and cellular senescence [1,2]. These processes contribute not only to functional decline but also to the onset of age-associated diseases, including cardiovascular disorders, neurodegeneration, metabolic syndromes, and skin photoaging [3]. Emerging environmental factors, such as chronic ultraviolet or blue-light exposure, may accelerate aging via overlapping mechanisms, including oxidative stress and inflammation, highlighting the need to consider both intrinsic and extrinsic contributors in anti-aging strategies [4]. Over the past two decades, natural products have emerged as a pivotal research domain for anti-aging interventions. Their intrinsic chemical diversity, spanning polyphenols, terpenoids, alkaloids, polysaccharides, peptides, and marine-derived metabolites, provides a wealth of bioactive scaffolds capable of targeting multiple aging-related pathways simultaneously [5,6,7]. In parallel, synergy-focused combination strategies, AI-assisted mechanism discovery such as molecular docking with neural-network scoring, and green extraction-to-formulation pipelines are increasingly used to enhance efficacy, exposure, and translational readiness [8,9]. Current scientific efforts therefore focus on elucidating mechanisms and translating these insights into preventive, therapeutic, and cosmetic applications, with attention to standardization, quality control, and clinically relevant outcomes.

## 2. Mechanistic Insights into Anti-Aging Effects of Natural Products

Recent studies demonstrate that natural compounds, including polyphenols, terpenoids, flavonoids, and alkaloids, modulate multiple signaling pathways intricately involved in the aging process [10] (see Table 1). For example, polyphenols such as epigallocatechin gallate (EGCG) and resveratrol activate the Nrf2/ARE pathway, enhancing the expression of antioxidant enzymes such as heme oxygenase-1 (HO-1) and glutathione peroxidase, thereby strengthening cellular defenses against oxidative stress [11]. Meanwhile, flavonoids, terpenoids, and alkaloids can attenuate chronic, low-grade inflammation (so called ‘inflammaging’) by suppressing NF-κB, MAPK, and JAK/STAT signaling, reducing the production of proinflammatory cytokines such as TNF-α, IL-1β, and IL-6 [12]. Moreover, compounds including ginsenosides and quercetin support mitochondrial homeostasis by stimulating AMPK/PGC-1α signaling, promoting mitochondrial biogenesis and energy metabolism [13]. Curcumin, spermidine, and berberine help maintain cellular health by regulating autophagy and ensuring proper protein folding and clearance, acting through mTOR, SIRT1, and FOXO pathways [14,15,16,17]. Nordihydroguaiaretic acid increased median lifespan in male UM-HET3 mice under rigorous, multi-site testing, supporting the longevity-promoting potential of antioxidant natural products [18]. These actions maintain cellular homeostasis and protect protein integrity, both of which are crucial for slowing age-related decline. Collectively, these multitarget activities underscore the advantages of natural products in counteracting the multifactorial processes of aging compared with single-target synthetic agents.

## 3. Synergy Strategies from Pairwise Targets to Multicomponent Formulations

Practical synergy can be achieved by combining agents that act at complementary points within or across pathways and by optimizing mixing ratios. First, glyceollin plus luteolin synergistically inhibits α-glucosidase; the 3:7 ratio yields a combination index of 0.642, with glyceollin showing competitive inhibition and luteolin a mixed mode, exemplifying complementary enzyme-level mechanisms [19]. Second, a two-extract blend of *Rhynchosia nulubilis* and *Polygonum multiflorum* promotes human dermal papilla cell proliferation under testosterone stress, with synergy most evident at 4:1 and accompanied by selective increases in IGFBP-1 and NT-3, indicating a growth factor-mediated tissue level mechanism [20]. Third, while TADIOS (a 1:1:1 ratio mixture of *Taraxacum officinale*, *Dioscorea batatas*, and *Schizonepeta tenuifolia*), whose name derives from the three genera (Ta-Dio-S), was originally investigated in respiratory disease, the rationale for this tri-herbal formulation lies in its ability to suppress molecular inflammation and enhance cellular resilience. Since chronic low-grade inflammation is a central driver of aging, the mechanistic complementarity of TADIOS provides a representative example of how natural products can be combined to achieve anti-aging benefits. Notably, in head-to-head tests, this mixture outperformed any single extract [21], underscoring the importance of synergistic strategies. Overall, from enzyme-level pairs to two-extract blends and equal part tri-herbal formulations, simple ratio tuning and complementary actions provide a practical route to synergy.

## 4. Mechanistic Discovery with Molecular Docking and Neural Networks

Combining molecular docking with artificial neural networks enables practical, hypothesis-driven exploration of natural-product mechanisms. Docking enumerates plausible ligand–target interactions for aging-relevant proteins, and Artificial Neural Network (ANN)-based scoring refines pose selection and affinity ranking to deliver actionable shortlists for experimentation. As one example, optimized ultrasound-assisted extraction of *Allium sativum* leaves yielded fractions with preserved antioxidant activity. A RSM (response surface methodology)-ANN-GA (genetic algorithm) workflow improved yield and potency metrics, illustrating how data-driven extraction pairs with mechanism-focused docking [22]. When paired with focused experiments, this approach can reduce time and cost while preserving biological interpretability. In this context, Convolutional Neural Network (CNN)-based scorers available in GNINA 1.3 have been reported to outperform classical scoring functions in structure-based virtual screening [23].

## 5. Process Optimization from Extraction to Formulation

Even with promising early results, natural products often show low absorption and large variation in their active compounds. To overcome this, improving both the production and formulation steps is essential. Green extraction methods such as deep eutectic solvents (DESs) can boost yields and better preserve active compounds, sometimes working better than regular solvents. RSM helps predict and improve extraction results, while ANNs [24] add accuracy when results are complex; together they quickly adjust the solvent mixture, temperature, and ultrasound to maximize bioactivity [22,25]. In later stages, better formulations can improve absorption. For example, a Box-Behnken design has been used to optimize nanostructured lipid carriers for a plant flavonoid (quercetin), leading to higher skin delivery [26]. Overall, linking extraction with formulation provides a clear path toward standardized nutraceuticals and therapies for aging-related conditions.

## 6. Conclusions and Outlook

Research on natural products for healthy aging is rapidly advancing, supported by innovations in green extraction such as DES and standardized synergy assessment. Mechanistic studies increasingly move beyond phenotypic readouts by mapping compound interactions with enzymes and protein targets involved in autophagy, redox balance, and inflammatory signaling. In parallel, AI- and machine learning-driven approaches are emerging to predict compound-target interactions and optimize bioactive combinations, although challenges remain regarding data availability, interpretability, and bias. Addressing these limitations through transparent algorithms and integration with experimental validation will be essential to realize translational potential.

In alignment with this Special Issue, future progress will depend on rigorous compositional verification such as HPLC, LC-MS/MS, NMR, robust dose-response characterization, clear synergy metrics, appropriate controls, and safety evaluation relevant to older adults. This Special Issue aims to bring together advances in chemistry, biology, and translational science to foster mechanism-informed and reproducible development of natural products for healthy aging. We welcome contributions that deepen mechanistic insight, strengthen reproducibility, and move the field toward clinically meaningful applications.

## Figures and Tables

**Table 1:** Representative classes of natural products and their molecular targets/mechanisms in aging-related pathways.

Natural Product Class	Representative Compounds	Key Molecular Targets	Mechanistic Outcomes
Polyphenols	Resveratrol, Quercetin, EGCG	SIRT1, AMPK, NF-κB, mTOR	Autophagy **Up**, Antioxidant defense **Up**, Inflammation **Down**
Terpenoids	Ginsenosides, Astragaloside IV	PI3K/AKT, MAPK, Nrf2/ARE	Neuroprotection **Up**, Redox balance **Up**
Alkaloids	Berberine, Caffeine	AMPK, JAK/STAT	Mitochondrial function **Up**, Pro-inflammatory signaling **Down**
Polysaccharides	β-glucan, Fucoidan	TLR4/NF-κB, MAPK	Immune modulation **Up**, Chronic inflammation **Down**
Peptides	Collagen peptides, Casein-derived peptides	mTOR, IGF-1	Proteostasis **Up**, Tissue regeneration **Up**
Marine metabolites	Fucoxanthin, Astaxanthin	Nrf2, FOXO, SIRT1	Antioxidant response **Up**, Cellular senescence **Down**

The bold font (Up or Down) indicates that the expression of key molecular targets is enhanced, reflecting the promotion of the corresponding phenomenon.

## Data Availability

No new data were created or analyzed in this study. Data sharing is not applicable to this article.
